# Compassion fatigue as bruises in the soul: A qualitative study on nurses

**DOI:** 10.1177/09697330211003215

**Published:** 2021-07-20

**Authors:** Tove Gustafsson, Jessica Hemberg

**Affiliations:** Åbo Akademi University, Finland

**Keywords:** Compassion fatigue, experiences, interviews, nurses

## Abstract

**Background:**

Nurses who are constantly being exposed to patients’ suffering can lead to compassion fatigue. There is a gap in the latest research regarding nurses’ experiences of compassion fatigue. Little is known about how compassion fatigue affects the nurse as a person, and indications of how it affects the profession are scarce.

**Aim:**

The aim of this study was to explore compassion fatigue experienced by nurses and how it affects them as persons and professionals.

**Research design, participants, and research context:**

A qualitative explorative approach was used. The data consisted of texts from interviews with seven nurses in various nursing contexts. Content analysis was used.

**Ethical consideration:**

Ethical approval was sought and granted from an ethics committee at the university where the researchers were based, and written, informed consent was obtained from all the participants.

**Findings:**

Five themes were discovered: Compassion as an empathic gift and compassion fatigue as a result of compassion overload, Compassion fatigue as exhausting the nurse as a professional and private person, Compassion fatigue as a crisis with potentially valuable insights, Compassion fatigue can be handled by self-care and focus on self, and Compassion fatigue is affected by life itself and multifaceted factors.

**Discussion:**

Compassion stress and overload can lead to compassion fatigue. Compassion fatigue affects the nurse’s ability to compassion, and the caring is no longer experienced in the same way; the nurses experienced it as being deprived of the gift of compassion. Compassion fatigue implicates a crisis with potentially valuable insights.

**Conclusion:**

Compassion fatigue can be symbolized as bruises in the soul, hurtful, but with time it can fade away, although it leaves a sense of caution within the nurse, which can affect the suffering patient.

## Introduction

Nurse ethical conduct is guided worldwide by the International Council of Nurses (ICN) Code of Ethics for Nurses, where compassion is one of five demanded professional values.^1^ Compassion is the heart of caring and consists of both being and doing.^2^ Compassion permeated by an inner ethos empowers nurses to act toward alleviating suffering and guides the mind, the hand, and the heart as the will to do what is good.^2^ Compassion is often defined as a “moral emotion” required for excellent nursing,^3^ or as a virtue which, according to Newham et al.,^4^ can be seen as a perception of suffering with the motivation to alleviate it.^5,6^ This is because the suffering of others touches, and thus motivates, a person to relieve it.^5,6^ This may be compared to “seeing by feeling” which, according to Jacobson,^7^ is what happens when encountering compassion. Newham et al.^4^ similarly describe compassion as being moved, understood as both affected and motivated, by the suffering of another and responding in practical ways. Compassion is also portrayed as behaviors and actions of empathy, kindness, patience, of offering hope and comforting.^4^ In order to be caring and compassionate, Newham et al.^4^ have found that it is necessary to see other persons as they truly are, which resonates with the ideas of nurses being morally perceptive and sensitive to patients.^8–10^ Gallagher^11^ stresses that it is admirable if health professionals are able to find opportunities to demonstrate kindness and compassion even in busy care contexts.

Ethics provides us with the tools to think critically in order to improve the ethical aspects of practice.^11^ However, caring about the patient is perceived to be lacking in some National Health Service hospitals in the United Kingdom.^12,13^ Even though nurses are taught about ethics, reports still indicate that physical tasks are claimed to every so often be performed without emotional engagement or recognition of patients’ need to feel cared about.^13^ Newham et al.^4^ claim that failures in compassionate care can occur if caring is reduced to technical tasks thus losing the moral, compassionate component of nursing, even if these tasks are performed competently. Gallagher,^11^ however, underlines that it is not difficult to imagine practitioners who think they show compassion but lack a sense of social justice, courage, and respect for individuals. She also stresses that we cannot expect too much from caregivers with regard to compassion, and that all care shortfalls cannot be ascribed to a lack of compassion in a person, since things can go wrong in different ways and for diverse reasons such as pathological work cultures or poor leadership.^11^ The aim of this study was to explore compassion fatigue experienced by nurses and how it affects them as persons and professionals.

## Background

Satisfaction arises when nurses experience positive feelings of caring compassion.^14^ Compassion satisfaction is constantly challenged by varying demands on nurses, and constantly being exposed to patients’ suffering, regardless of how satisfactory the outcome is, can lead to *compassion fatigue*.^14^ Compassion fatigue is characterized by an inability to cope with the emotional stress caused by the long-term exposure to suffering people and leads to emotional, physical, and spiritual exhaustion.^15^ Compassion fatigue is a complex concept that has been honed for the past 30 years, and it originated in caregivers’ post-traumatic stress syndrome from which it has crystallized into secondary trauma and further into the concept of compassion fatigue with Charles Figley at the forefront.^15^ Carla Joinson^16^ coined the concept of compassion fatigue in 1992 and defined it as a unique form of fatigue affecting healthcare professions. Coetzee and Laschinger^17^ have investigated the various theories of compassion fatigue and conclude that all are based on Figley’s^18^ theory in which the definition of compassion fatigue is “a state of exhaustion and dysfunction, biologically, physiologically, and emotionally, as a result of prolonged exposure to compassion stress” (p. 34). In addition, compassion fatigue is explained as a phenomenon that can occur when the amount of compassion spent exceeds the capacity to cope or recover.^19^ Thus, it can be concluded that compassion fatigue is an ethical question—if suffering from compassion fatigue, the nurse is prevented from following a nurse’s inner ethical desire of compassion and willingness to do good for the patient.

Situations that trig compassion fatigue include unkind or biased behavior from patients or their families, patient suffering, colleagues’ abusive attitude toward patients, death of a patient, failed resuscitation, mistakes,^20^ deterioration of patients’ physical health, bad news for patients, and suffering caused by treatment or patients’ emotional conflicts.^21^

Previous research indicates conflicting results in relation to socio-demographic risk factors for compassion fatigue, and some studies propose that *females* have increased risk,^22^ while others suggest the opposite^23^ or find no difference.^24^ The same applies for *marital status*: according to some studies, married nurses have increased risk for compassion fatigue^23,25^ while the results from Chen et al.^26^ show the opposite. Most studies agree that *younger age* and *little work experience* are risk factors for compassion fatigue^27–33^ but there are also contradictive results.^25–27^ Higher levels of *education* are further known to increase the risk of compassion fatigue.^24,27,28^ *Economic status* is found to affect compassion fatigue in two ways: difficulties increase the risk^34^ or it has no impact.^23^ Person-related risk factors are *health problems*,^34,35^ *deficient coping-strategies*,^36,37^ *low degree of resilience*,^28^ *problems with self-esteem*,^38^ and *dealing with emotions*.^38,39^ Personality traits such as *hypersensitivity*, *introversion*,^39^ *neuroticism*,^37^ and *trait-negative affect* in general^39^ have also been shown to increase the risk of compassion fatigue. Organizational risk factors are as follows: increasingly higher *demands* from many different directions, increasingly tight *time constraints*,^31,34,36,40^ *lack of social support*,^36^ *high workload*,^31,32,34,35,40,41^ *constant process changes*, *too low staffing levels* and *lack of resources*,^31,32,42^ *work relations*, *lack of control*,^32^ and *insufficient leadership*.^41^ Contradicting outcomes exist regarding the connection between *shift work* and compassion fatigue.^24,25^

As compassion fatigue moves beyond the delicate boundary of being a normal reaction to a pathological condition, it takes on various expressions. Physical symptoms such as *fatigue*,^35,41,42^ *aches* and *pain*,^35,41^ *sleeping problems*,^32,36,41^ *gastrointestinal problems*,^32,41^ *impaired immune system*, and *exhaustion*^32^ have been found, as have mental symptoms including *emotional exhaustion*,^32,42^ *insensibility* and *decreased ability to empathize*, *cynicism*, *helplessness*,^32^ *depression*, *worry*,^32,42^ and *distancing*.^32,36^ Compassion fatigue has also been shown to lead to behavioral expressions such as *increased substance use*,^24,32^ *irritability*, *anger*, *avoidance of patients*,^32,41^ *impaired clinical decision making*, *absence*, *tension in relations*, and *compromised patient care*.^32^

Compassion fatigue has been studied in various contexts and is found in several areas of healthcare; acute, critical, and intensive care^25,28,29,31^; pediatrics^27^; psychiatry^23,24,43^; and in the care of older people.^30,44^ Ruiz-Fernandez et al.^25^ have concluded that compassion fatigue is to a greater extent found in primary care and in cities than in hospitals and rural areas.

Only scant research on treatment methods for compassion fatigue exists, but intervention studies with various outcomes are found.^39,42,45^ The research is unambiguous when it comes to the prevention of compassion fatigue, considered as the most important approach in fighting it.^28,30,31,36,46,47^ Some studies suggest that by increasing compassion satisfaction, the risk of compassion fatigue decreases.^29,39,46,48,49^ Other studies allocate preventive actions to personal and organizational actions. Personal actions such as *increased self-compassion*,^50^ *better work–life balance*,^31,35,36,39^ *healthy living*,^46^ *social relations*,^40,50^ and *activities*^32,35,36,47^ can prevent compassion fatigue. Furthermore, organizational actions such as *increased awareness* and *knowledge of compassion fatigue*,^36^ *tools to reduce stress during work*,^51,52^ *informal and formal peer support*,^41,50^ *increased influence*,^24,50^ *good leadership*,^29,34^ *strategies to distinguish between professional and personal roles*,^35,40^ and *ergonomic aspects*^36,50^ can prevent compassion fatigue.

Clearly, the phenomenon of compassion fatigue is studied, but little is known regarding the experience of compassion fatigue and its impact on nurses’ profession and particularly on the person. In order to comprehend the experience of compassion fatigue and its impact on nurses’ profession and their person, we applied Eriksson’s theory of caritative caring as the theoretical framework for this study because this theory penetrates deeper into the dimensions of a human being’s existence and subjective experiences; consequently, Eriksson’s theory of caritative caring was seen as a potential for supporting our quest for pursuing a profound understanding of this human phenomenon.^2,53^ Compassion is viewed as a core concept in caring science and is in the caritative caring theory seen as the motive of caring.^2^ In order to fully understand compassion fatigue, it is necessary to grasp compassion. From a caring science perspective, neither sympathy nor empathy are sufficient synonyms for compassion or even sufficient to describe it.^54^ Compassion is the most precious asset of caring and consists of both actions and presence. Compassion is ethical conduct that mediates solidarity, commitment, and accessibility with the human at the center and treated with dignity. Compassionate care can enhance health.^2^ Compassionate caring requires courage to approach suffering expressed as pain, devastation, confusion, anguish, sorrow, apathy, powerlessness, and anger, to name a few.^55^ Compassion is a merciful act^2^ and is also seen as a personality trait which implies that it is a virtue, in other words, more than correctness or good behavior.^55^ Compassion mediated by nurses can, according to [Hemberg and Wiklund Gustin, 2020],^56^ be seen as a belonging between the nurse and the patient in a form of natural human-to-human-connection, and this is the element that unites and holds the caring relationship together. Compassion is demanding as it requires strength to face one’s own emotions that arise from encountering suffering. Self-compassion is the confirmation of and reconciliation with one’s own vulnerability and suffering.^2^

Research shows that compassion fatigue has consequences for patients, nurses, and the entire healthcare system.^57^ Because of the organizational impact it has, it is also an economic issue.^58,59^ The world is changing at a record pace and with it also the caring reality. Consequently, there is a gap in the latest research regarding nurses’ experiences of compassion fatigue^60^: little is known about how compassion fatigue affects the nurse as a person, and indications of how it impacts the profession are scarce.

## Aims

The aim of this study was to explore compassion fatigue experienced by nurses and how it affects them as persons and professionals. The research question was as follows: How does nurses experience compassion fatigue and how does it affect them as persons and professionals?

## Methodological aspects

A qualitative explorative design was used for this study. The data were comprised of text from interviews with seven nurses in Finland about their experiences of compassion fatigue. Participant recruitment were made through social media (Facebook) and self-selection. A flyer was sent out through a personal Facebook page with information about the study and the possibility to participate in an interview regarding compassion fatigue. An invitation with the flyer as an appendix was also sent to all primacy care units in a region in Southern Finland. The inclusion criteria were working as a registered nurse and having personal experiences of suffering from compassion fatigue as well as being interested in sharing these experiences. Those nurses who were interested in participating could contact the researcher based on the contact information given on the flyer. The age of the participants varied between 29 and 57 years. Their worktime experience varied from 1 to 35 years of experience in various nursing contexts. The nurses included in this study worked in different contexts, for example, in emergency care, pediatrics, internal medicine, surgery, occupational nursing, and psychiatry. The participants knew from before what compassion fatigue is; however, the researcher also gave an introduction before the interview in order to clarify the meaning of the concept.

Semi-structured interviews were conducted both face to face, in the hospital organizations where the nurses worked/in the nurses’ own homes, and, due to the pandemic, by videoconference by the first researcher in May 2020. Each interview lasted about 45 min. All interviews were recorded and transcribed.

### Data material, data collection, and data analysis

All interviews were transcribed verbatim by the first researcher and all personal information was replaced with codes. Analysis was conducted using content analysis by Graneheim and Lundman.^61^ The researcher paid attention to the latent content of the data, the meaning when interpreting the text.^61^ The data were read and re-read, then analyzed to reveal meaning units. The meaning units were then condensed, coded, and placed in sub-categories and categories in order to find themes that described the meaning of the data.^61^ For an example of the data analysis, see Table 1.

**Table 1.** Example of the data analysis.

Meaning unit	Condensed meaning unit	Code	Category	Main theme
Compassion fatigue made the nurse tired	Fatigue	Compassion fatigue is a different kind of tiredness	Compassion fatigue affects health	Compassion fatigue as exhausting the nurse as a professional and as a private person
Fatigue that does not go away with sleep	Prolonged fatigue			
Prolonged fatigue that does not go away during a normal vacation				
A different kind of fatigue; cloudy, deep, sweeping. Like being in a cloud or tired from hangover	Deeper and different kind of tiredness			
Compassion fatigue leads to social life suffering	Loneliness	Compassion fatigue means social exclusion		
Compassion fatigue leads to distancing oneself from activities	Infirmity			
Compassion fatigue leads to withdrawing from contacting people				

### Ethical considerations

The study followed the guidelines of the Finnish Advisory Board on Research Integrity.^62,63^ Ethical approval was sought from an ethical committee at the university where the researchers were based and was granted 14 May 2020. Written, informed consent was obtained from all the participants. If the interview aroused unpleasant feelings in participants and they needed help to cope with this, they could contact the interviewer afterward.

## Findings

**Figure 1.**
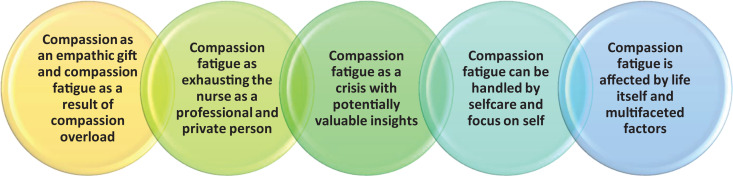
Study findings.

The results of this study generated five themes (see Figure 1). The five themes were “Compassion as an empathic gift and compassion fatigue as a result of compassion overload,” “Compassion fatigue as exhausting the nurse as a professional and private person,” “Compassion fatigue as a crisis with potentially valuable insights,” “Compassion fatigue can be handled by self-care and focus on self,” and “Compassion fatigue is affected by life itself and multifaceted factors.” The themes are somewhat interwoven with each other, for instance, the themes “Compassion fatigue as exhausting the nurse as a professional and private person” and the theme “Compassion fatigue as a crisis with potentially valuable insights” overlap. The themes are further described in the following.

### Compassion as an empathic gift and compassion fatigue as a result of compassion overload

Based on the nurses’ statements, the ability for compassion is seen as an empathic gift. In this study, compassion fatigue occurred as a result of exposure to patients’ constant suffering which led to compassion stress and overload. There were also occasions where the exposure was not constant, but nevertheless compassion fatigue occurred more suddenly. Common to both is the prerequisite/ability to compassion.

The fact that nurses are affected by the patient’s suffering makes them want to understand and care. This is what one participant said: “I am touched, like I want to help and I want to understand and I want to support…” (P3)

According to the nurses, the ability to compassion is not something learned, but something innate. It is seen as an empathic gift and thus something positive. One of the participants expressed it as follows: “The ability to compassion…or gift…a gift or a quality…that I have always had.” (P1)

However, the nurses also expressed that high exposure and overload of situations that require great empathic energy and compassion eventually could lead to compassion fatigue. One nurse put it like this:

There could be three in one day that came for a health examination and I only got to ask the first question “How are you?” and it was like turning on a tap…people broke down…the time allotted for the appointment was not nearly enough. (P4)

### Compassion fatigue as exhausting the nurse as a professional and private person

Compassion fatigue leaves its mark on the nurse both professionally and as a person. Regarding the profession, the nurses experience compassion fatigue as being deprived of the gift of compassion. It emerges as a feeling of indifference and meeting the patient can feel both inauthentic and repugnant. Compassion fatigue can also create an inability to face a suffering person. Nurses may have difficulties accessing their own feelings. One nurse put it this way: “Even less would I be able to meet someone…feel something for someone else…” (P1)

Compassion fatigue may give rise to many different emotions in the nurse both in the profession and as a person. These are mainly negative emotions such as guilt, shame, sadness, irritation, impatience, inadequacy, and increased fear. One participant said, “So it got a little shameful that I got so tired myself…I have felt bad because of this, I think I take it out on the wrong people [family members].” (P4)

One participant explained how she tried to maintain her professionalism and manage the job as a nurse: “…one had to control oneself and treat the other as a patient.” (P3)

Another nurse described the feelings of inadequacy that arose from compassion fatigue:

I was not worth being at work…my patients have not deserved such a bad nurse as I am…who does not seem to be able to be there and cannot care enough and like have to go home…it was probably a kind of grief as well. (P2)

The result shows that compassion fatigue affects the nurse’s health. Compassion fatigue is described as a different kind of fatigue that completely drains the individual of energy and that leads to physical, mental, and behavioral problems. This is how one participant expressed it: “It was a deeper fatigue…which seemed to lie in the soul…it weighed on my soul.” (P4)

Compassion fatigue gave rise to headaches and ill-being in the form of nervousness, anxiety, distress, and tearfulness. The fatigue is described as heavy as lead, as some kind of brain fatigue and as a fatigue that does not go away by sleeping.

A very deep fatigue, it was like cloudy fatigue…like inside the head…a misty fatigue, a different fatigue…like you are you are inside a cloud in your head…like a fatigue from a hangover but worse…” (P3)

Furthermore, health is affected by compassion fatigue in that it leads to loneliness and lack of energy. It also affects those closest to the nurse. These feelings within the person need to be processed and have an outlet and, therefore, the nurses often distance themselves from activities and live out the feelings at home. All participants said that compassion fatigue first manifested itself during leisure time before it became too heavy affecting work. This may indicate a high work ethic among the nurses as they put their profession first, although the compassion fatigue had taken almost all their energy. This is how one nurse put it: “While you are at work, you hold yourself together…but then when the workday is over you are completely empty” (P3).

Compassion fatigue is experienced as changes in the professional self. These changes manifest as taking on a shield to protect the self from feeling compassionate and as emotions that have arisen at work but are kept to oneself. The changes are also felt as something missing in the profession, leading to action-centered care. One participant said, “I’m a bit like a kind of foil-clad person, in some way it bounces…what is said does not reach me all the way…it bounces back somehow” (P5).

Compassion fatigue has consequences for the profession in the form of absenteeism. The nurses stated that if they had at some point magnified a symptom they had to be able to take a few days off to have the opportunity to ponder it themselves.

### Compassion fatigue as a crisis with potentially valuable insights

Compassion fatigue leads to a crisis for the nurse, both in terms of the professional role and as a private person. The nurse wonders if compassion fatigue is too high a price to pay for a job and feels anxious about the future. One participant put it this way: “I sometimes wonder if it’s [the work] worth it…has it been worth it [the compassion fatigue]?” (P7).

The experience of compassion fatigue can lead to the nurse gaining new insights, learning self-compassion and to an inner growth as a human being. This is what one of the participants said, “It has after all made me more…humble…I actually have to stop and try to listen to myself from time to time…” (P2)

Another participant says that compassion fatigue led to a change of profession: “I then came to the conclusion that I was not in the right place either…as a nurse in the field…I do not think I will work as a nurse again.” (P2)

### Compassion fatigue can be handled by self-care and focus on self

A healthy lifestyle, time for reflection, recovery, and social networking are important aspects in order for the nurse not to end up in chronic compassion fatigue. A balance between rest and exercise and reflection in the form of informal or professional support as well as a feeling that there is a deeper meaning or purpose supports the nurse on the way back from compassion fatigue. This is how one participant describes how to heal with the help of self-care: “It has felt like you have bruises in your soul but that it disappears with time when you take care of yourself.” (P3)

Focusing on one’s inner convictions or religious beliefs can serve as support to deal with compassion fatigue. One participant said the following: “I have a faith that I feel has…has done…that I have been able to bear it and I have been able to face these very heavy feelings myself and still continue…” (P1)

### Compassion fatigue is affected by life itself and multifaceted factors

The onset of compassion fatigue is affected by certain personality traits such as being a brooding type and an emotional person. Compassion fatigue can also more easily arise if a person has high demands on self and thus has difficulty being self-forgiving. In addition, the onset of compassion fatigue can be affected if at the time there were other factors in the person’s life that took much energy, for instance, one’s own state of health, dissatisfaction with the place of residency, or the busy years in life. This is how one participant put it: “I did not feel so good…So much happened during that time that I couldn’t catch up with.” (P1)

Working conditions can also affect the onset of compassion fatigue. Uneven distribution of tasks, injustice, and insufficient support are examples of such factors that the leader can influence. One participant described what a harsh work climate might look like: “If you feel something [feelings arise because of being compassionate], you are in the wrong profession…I heard that from my previous employer.” (P1)

Insufficient collegial support can also contribute to the development of compassion fatigue. This is what one participant said: “As I may now miss that…you have such a feeling of we-spirit…it is also collegial support” (P5).

## Discussion

The aim of this study was to gain a deeper understanding of how compassion fatigue is experienced by nurses and how it affects them. The analysis generated five themes.

This study found that the ability to compassion is something that is required for compassion fatigue to occur. This is in line with Figley’s^18^ theory, that the ability to compassion is the basis of compassion fatigue. Duarte and Pinto-Gouveia^38^ claim that beyond a certain level of compassion, there is a risk of compassion fatigue. This study also found that nurses see the ability to compassion as something positive and as an emphatic gift, an innate quality rather than a learned method. This is in line with Eriksson’s caritative theory.^2^ Bond et al.^64^ state that the word compassion refers to a natural characteristic or attribute that cannot be taught but claim that it can be developed through repetition of behavior that is observed in practice.

However, this study found that compassion fatigue does leave a mark in the nurse both as a person and as a professional. Compassion fatigue affects the nurse’s ability to compassion and the caring is no longer experienced in the same way; the nurses experienced it as being deprived of the gift of compassion. This is in line with Dekeseredy et al.^65^ and Finley and Sheppard,^40^ where it emerged that nurses who experienced compassion fatigue distanced themselves from patients’ they knew needed emotional support. Dekeseredy et al.^65^ also conclude that the nurses’ behavior became tougher and they were more cynical in their approach as a way for them to protect themselves from emotional situations. The same phenomenon is found in this study where nurses experience compassion fatigue as wearing a shield of protection against becoming emotionally engaged. In this study, compassion fatigue is experienced as giving rise to different emotions, usually negative ones. The nurses felt they were insufficient and not authentic in meeting the suffering patient. This is supported by Finley et al.^40^ who have found that nurses felt feelings of guilt over their compassion fatigue. Duarte et al.^38^ reveal that nurses who avoid confronting negative emotions to a greater extent risk compassion fatigue.

Compassion fatigue can have consequences for the profession.^29,58^ According to Kelly and Lefton,^29^ a significant proportion of nurses who experience compassion fatigue have had thoughts of changing profession at some point during their careers. Yang and Kim,^20^ on the contrary, have found no connection, either directly or indirectly, between compassion fatigue and the idea of changing workplace or profession. The results from this study show that nurses both leave the profession and have increased absenteeism. Increased absence due to compassion fatigue is also found in Sinclair et al.^32^

This study shows that the energy that remained when the nurses experienced compassion fatigue was used primarily for work. They experienced that they managed to get through the working day, but that their leisure time was affected by the fatigue, and this led to withdrawing from activities and social life suffering. Finley and Sheppard^40^ have found that nurses suffering from compassion fatigue are so exhausted after work that they isolate themselves. The results of this study show that the nurses experienced guilt over the family. This is supported by both Dekeseredy et al.^65^ and Wentzel et al.,^42^ compassion fatigue had a negative effect on the family in their studies as well.

This study also reveals that compassion fatigue affect nurses’ health and they reported physical, mental, and behavioral problems arising from compassion fatigue. The fatigue that the nurses experienced was not any ordinary fatigue but deep and sweeping and it could not be reversed by sleeping. Nolte et al.^36^ call this fatigue as being worn out and Wentzel et al.^42^ describe it as not only physical but emotional as well.

Another finding in this study was that compassion fatigue implicates a crisis with potentially valuable insights. This crisis affects the nurse both in the professional role and as a private person. The participants expressed doubts about their profession and worries about the future. Some participants in the study had thought about changing workplace or leaving the profession and some had left the profession. Similarly, Nimmo and Huggard^66^ have found that compassion fatigue can lead to workforce dropout. Peters^57^ has found that compassion fatigue leads to doubts about one’s own values and thoughts about leaving the profession. Fukumori et al.^67^ show that nurses who experience compassion fatigue can reflect on why they have chosen the profession. Wentzel et al.^33^ state that over time, the nurse who has experienced compassion fatigue becomes stronger, which supports the results from this study where it appears that compassion fatigue can provide new experiences and support personal growth. In other words, a nurse who has experienced compassion fatigue can more easily recognize symptoms and, as Wiklund Gustin^68^ claims, it is necessary to react on early symptoms and see them as an alarm system.

The results of this study show that a focus on self and self-care can help with compassion fatigue and highlight what helped nurses recover from compassion fatigue: healthy living habits, time to reflect, and support from a social network. Nolte et al.^36^ indicate that exercise, reflection, social relationships, and focusing on a spiritual outlet help with recovery. This study also shows that a religious belief can be helpful.

In addition, the present study reveals that the onset of compassion fatigue is affected by life itself and multifaceted factors. This finding is in line with Durkin et al.^49^ who have discovered that people with higher demands on themselves have difficulties being self-forgiving and thus have a greater risk for compassion fatigue. In this study, certain personality traits appear to be potential causes of compassion fatigue. Craig and Sprang^39^ point to explicitly negative personality traits as a risk, while the results from this study suggest qualities such as being an emotional person or a thinker. Duarte et al.^69^ argue that a tendency to be self-critical is a risk, which supports the results of this study. Duarte and Pinto-Gouveia^38^ have also found that nurses that are more self-judgmental and have less psychological flexibility are more disposed to compassion fatigue. The nurses in this study stated that the time in life and their own health state at the moment of compassion fatigue can affect the onset of it. This is supported by Figley^18^ who refers to “other life demands”: in other words, the onset of compassion fatigue is affected by other demands currently put on the nurse. The results of this study point to factors in the work organization and work community, as well as compassion overload due to high exposures of patient suffering and, thus, situations that require great empathic energy and compassion are possible causes of the development of compassion fatigue. Mottaghi et al.^70^ have likewise found that empathy as well as secondary traumatic stress could explain some of the links between clinical empathy and symptoms of compassion fatigue. This is also in line with Nolte et al.^36^ who say causes of compassion fatigue can be found in the work environment. Both Chen et al.^26^ and Balinbin et al.^71^ point out that strategies that promote collegial relations are needed. Instead of putting out fires, the management should invest preventively and proactively to reduce the risk of compassion fatigue.^29^ The results from this study show that there can be a harsh climate in the workplace, and it is supported by Brint^72^ who describes how a stoic culture prevails in many workplaces. In addition, we would suggest *group sessions* where nurses can share and exchange their experiences of stressful situations requiring their empathetic abilities in order to support their anxiety reduction and increase their sense of empowerment,^73^ which, ultimately, may help to reduce the risk of compassion fatigue. In addition, we propose a balanced approach to caring for patients, as well as a sufficiently relieving work environment, particularly designed for caregivers who are highly exposed to compassion stress, involving flexible work routines and staffing, and longer periods of time off between work shifts to enable necessary time for reflection and recovery.

### Strengths and limitations

One limitation to this study might be that there were only female participants and had there been male ones, the findings might have differed. In order to strengthen the study’s credibility, an effort has been made to respond to the demands of reliability, and transferability. As regards credibility in the selection of participants, all of them had experienced compassion fatigue. The number of participants is considered sufficient. Most of the participants had considerable worktime experience. Credibility in the analysis has been sought by verifying the analysis steps throughout in line with the method. Furthermore, the data analysis has been conducted in close collaboration with the second researcher, which is an experienced researcher in qualitative methods. The researchers discussed and agreed upon the final themes. Descriptive quotes have been used to respond to the requirements of reliability in the results. To strengthen reliability, an endeavor has been to provide a clear and comprehensive description of the methodological procedure. The goal of the pursuit of credibility was to obtain transferable results that can be applied in all areas of social and healthcare.

## Conclusion

Ability for compassion is seen as a natural attribute of the nurse and thus as an empathic gift, and high exposure and overload of patient suffering situations that require great empathic energy and compassion can lead to compassion fatigue. Compassion fatigue is a crisis for the nurse both as a person and as a professional. Compassion fatigue deprives the nurse from the gift of compassion and sets its marks on the ability to alleviate suffering. The health of the nurse is broadly affected by compassion fatigue and the fatigue is not only physical but also emotional and social. The fatigue weighs on the nurses’ soul. The leisure time is primarily affected because the nurse puts all available energy into the work. Compassion fatigue gives rise to negative emotions such as guilt, shame, and anger, and it has consequences both for the person and the profession. Compassion fatigue can be emblematic of a bruise in the nurse’s soul. A bruise is hurtful and leaves a mark though it fades away with time. To avoid bruises in the future, the nurse may be careful not to be hurt again. Yet, by being careful, the nurse’s ability to compassion may be affected and the nurse may not be able to alleviate suffering.
